# Structural and Sequence Analysis of Imelysin-Like Proteins Implicated in Bacterial Iron Uptake

**DOI:** 10.1371/journal.pone.0021875

**Published:** 2011-07-25

**Authors:** Qingping Xu, Neil D. Rawlings, Carol L. Farr, Hsiu-Ju Chiu, Joanna C. Grant, Lukasz Jaroszewski, Heath E. Klock, Mark W. Knuth, Mitchell D. Miller, Dana Weekes, Marc-André Elsliger, Ashley M. Deacon, Adam Godzik, Scott A. Lesley, Ian A. Wilson

**Affiliations:** 1 Joint Center for Structural Genomics, La Jolla, California, United States of America; 2 Stanford Synchrotron Radiation Lightsource, SLAC National Accelerator Laboratory, Menlo Park, California, United States of America; 3 Wellcome Trust Sanger Institute, Wellcome Trust Genome Campus, Hinxton, United Kingdom; 4 Department of Molecular Biology, The Scripps Research Institute, La Jolla, California, United States of America; 5 Protein Sciences Department, Genomics Institute of the Novartis Research Foundation, San Diego, California, United States of America; 6 Center for Research in Biological Systems, University of California San Diego, La Jolla, California, United States of America; 7 Program on Bioinformatics and Systems Biology, Sanford-Burnham Medical Research Institute, La Jolla, California, United States of America; University Paris Diderot-Paris 7, France

## Abstract

Imelysin-like proteins define a superfamily of bacterial proteins that are likely involved in iron uptake. Members of this superfamily were previously thought to be peptidases and were included in the MEROPS family M75. We determined the first crystal structures of two remotely related, imelysin-like proteins. The *Psychrobacter arcticus* structure was determined at 2.15 Å resolution and contains the canonical imelysin fold, while higher resolution structures from the gut bacteria *Bacteroides ovatus*, in two crystal forms (at 1.25 Å and 1.44 Å resolution), have a circularly permuted topology. Both structures are highly similar to each other despite low sequence similarity and circular permutation. The all-helical structure can be divided into two similar four-helix bundle domains. The overall structure and the GxHxxE motif region differ from known HxxE metallopeptidases, suggesting that imelysin-like proteins are not peptidases. A putative functional site is located at the domain interface. We have now organized the known homologous proteins into a superfamily, which can be separated into four families. These families share a similar functional site, but each has family-specific structural and sequence features. These results indicate that imelysin-like proteins have evolved from a common ancestor, and likely have a conserved function.

## Introduction

Iron is an essential element to almost all organisms. However, it is poorly soluble at physiological pH and toxic in the presence of O_2_. As a result, bacteria have evolved complex and diverse mechanisms for iron uptake and metabolism [1]. Most genes involved in iron acquisition are repressed by the Fur (ferric uptake regulation) transcription factor, such that they are expressed only when the level of free iron in the cell is low.

IrpA (iron regulated protein A) was previously found to be essential for growth under iron-deficient conditions in the cyanobacteria *Synechococcus sp.* (strain PCC7942) [2]. A few other homologous proteins in bacteria have a conserved role in iron uptake or metabolism as they are also regulated by Fur as, for example, in *Pseudomonas aeruginosa* [3] and *Vibrio cholera* [4]. An IrpA homolog from *P. aeruginosa* (PA4370) was characterized as a zinc peptidase [5], and was named imelysin (insulin-cleaving membrane protease, ICMP) due to its localization to the outer membrane and its ability to cleave insulin. ICMP contains an HxxE sequence motif that was previously observed in the MEROPS [6] metallopeptidase family M14; thus, ICMP was believed to bind zinc. ICMP served as the founding member of the M75 peptidase family in MEROPS. Besides involvement in iron uptake, imelysin homolog LruB from pathogenic *Leptospira interrogans* was also found to be involved in bacterial pathogenesis by playing a significant role in human equine recurrent uveitis [7], [8], probably due to its presence on the cell surface.

More recently, a new iron-transporter EfeUOB was characterized [9], [10]. This system is involved in Fe^2+^ transport at low pH conditions. EfeU is homologous to yeast iron permease Ftr1p, while EfeB is likely a periplasmic di-heme peroxidase (a member of DUF1111 protein family). EfeO is an essential component of the EfeUOB operon. However, its biochemical function is unknown. EfeO contains a C-terminal 250-residue imelysin-like domain and an N-terminal cupredoxin(CUP)-like domain that may bind iron [11]. The EfeUOB operon is highly conserved in bacteria [9], [11]. The genomic context of all previously characterized imelysins bears substantial similarity to EfeUOB, except that EfeU is absent in the operons that encode putative outer-membrane clusters. The *P. aeruginosa* Fur-regulated imelysin operon (PA4370–PA4373) consists of two imelysin-like proteins (PA4370/ICMP and PA4372), and one EfeB homolog (PA4371). Thus, imelysin-like proteins appear to have a conserved role in iron uptake.

Imelysin-like proteins are widely distributed in bacteria but poorly characterized and it remains to be elucidated how imelysin-like proteins function in iron transport. In order to gain further understanding of the structure and function of this family, we selected 24 imelysin-like proteins for structure determination at the Joint Center for Structural Genomics (JCSG, http://www.jcsg.org) [12], [13]. Here, we report two crystal structures of this novel protein family, a circularly permuted imelysin (with the GxHxxE motif) from *Bacteroides ovatus* (PIBO) and an imelysin-like protein (with a variant GxxxxE motif) from *Psychrobacter arcticus* 273-4 (IPPA). *P. arcticus* 273-4 was isolated from a sample derived from the 20,000 year-old (at least) Siberian permafrost core [14], while *B. ovatus* is a predominant member of human gut microbiome. Iron-uptake mechanisms in these bacteria are currently poorly studied. However, PIBO and IPPA are both located in putative operons that are similar in other bacteria, suggesting conserved roles in iron uptake where PIBO is functionally equivalent to ICMP (PA4370), and IPPA equivalent to PA4372 of *P. aeruginosa*. PIBO and IPPA adopt a structure distinctive from known metallopeptidases or iron-binding proteins.

## Results and Discussion

### Structural determination and model quality

The crystal structures were determined with the high-throughput structural genomics pipeline implemented at the JCSG [12], [13]. Full-length PIBO and IPPA contain 384 and 389 residues respectively. The N-termini of PIBO (^1^ MMKTKFFYVAALILGLAFTTTSC ^23^) and IPPA (^1^ MKINHVLAMALSALSAGILISC ^22^) match the lipoprotein signal peptide motif of Gram-negative bacteria that usually consists of one or more positive charged residues, followed by a stretch of hydrophobic residues and a lipobox motif containing an invariant cysteine [15]. To facilitate purification and crystallization, these predicted N-terminal lipoprotein signal peptides (residues 1–24 for PIBO; residues 1–26 for IPPA) were not included in the expression constructs. Selenomethionine derivatives of PIBO and IPPA were expressed in *Escherichia coli* with an N-terminal tobacco etch virus (TEV) cleavable His-tag and were purified by metal affinity chromatography. Crystals were obtained in various crystallization conditions and were harvested and screened for diffraction.

Two multiple-wavelength anomalous diffraction (MAD) datasets, corresponding to the best diffracting crystal of the two crystal forms of PIBO, were collected at Stanford Synchrotron Radiation Lightsource (SSRL) beamlines. Both datasets were indexed and processed in monoclinic space group C2; however, the two crystal lattices are not directly related. Structures for the two crystal forms were solved independently using the MAD phasing method. The initial experimental density maps (Fig. S1) and final refined maps were both of excellent quality. The structure from crystal form 1 (PDB code 3n8u) was refined to a resolution of 1.44 Å with an R_cryst_ of 16.7% and R_free_ of 19.0%. The model displays good geometry with an all-atom clash score of 2.98, and the Ramachandran plot produced by MolProbity [16] shows that all, but one (A/88), of the residues are in allowed regions, with 97.2% in favored regions. The side chains were also well defined, and were refined with only four rotamer outliers. The asymmetric unit (asu) of the final model contains two monomers (A, residues 33–384, and B, residues 31–384), 1191 water molecules and other solvent molecules that were present in the purification, crystallization or cryo-protection reagents, including three magnesium ions, two chloride ions, and seven ethylene glycol molecules. The structure from crystal form 2 (PDB code 3oyv) was refined to a resolution of 1.25 Å with an R_cryst_ of 13.1% and R_free_ of 16.3% and with good geometry similar to the first structure. The asu of the final model contains one monomer (A, residues 30–384), two chloride ions, two glycerol molecules, and 533 water molecules. The two monomers in the asu of crystal form 1 of PIBO are essentially identical with an rmsd of 0.5 Å for 352 Cα atoms. These two monomers are also very similar to the monomer in crystal form 2, except for a region between residues 329 and 365 (rmsd 4.0 Å overall, 0.63 Å if residues 329–365 are omitted). The second PIBO structure at higher resolution contains significantly more residues that display multiple conformations (57 for one monomer) than in crystal form 1 (45 for two monomers).

The IPPA structure was determined using MAD data collected at the Advanced Light Source (ALS). The data were indexed and processed in orthorhombic space group I222. The structure at 2.15 Å resolution (PDB code 3pf0) was refined to an R_cryst_ of 18.2% and R_free_ of 21.8%. The model also displays good geometry with an all-atom clash score of 3.41, and the Ramachandran plot produced shows that all residues are in allowed regions, with 96.8% in favored regions. The final model contains one monomer (A, residues 66–383) and 216 water molecules in the asu. The N-terminal region (residues 27–65) and C-terminal region (residues 384–389) were disordered and were not included in the final model. Data processing and refinement statistics are summarized in Table 1.

**Table 1:** Data collection, phasing and refinement statistics.

Protein	PIBO						IPPA		
Space group	C2						I222		
PDB ID	3n8u			3oyv			3pf0		
Unit cell	*a* = 131.5 Å, *b* = 49.9 Å, *c* = 128.3 Å, β = 117.6°			*a* = 104.4 Å, *b* = 47.9 Å, *c* = 83.9 Å, β = 124.6°			*a* = 60.7 Å, *b* = 69.1 Å, *c* = 165.1 Å		
Data collection	λ_1_ MADSe	λ_2_ MADSe	λ_3_ MADSe	λ_1_ MADSe	λ_2_ MADSe	λ_3_ MADSe	λ_1_ MADSe	λ_2_ MADSe	λ_3_ MADSe
Wavelength (Å)	0.9795	0.9184	0.9790	0.9793	0.8550	0.9791	0.9795	0.9537	0.9793
Resolution range (Å)	29.7–1.44	29.7–1.44	29.7–1.52	41.9–1.3	31.2–1.25	41.9–1.3	45.6–2.15	45.6–2.2	45.6–2.24
Number of observations	546,797	563,162	481,842	259,065	340,713	260,599	67,901	62,568	59,040
Number of unique reflections	132,077	132,203	112,658	75,095	93,405	75,312	19,285	17,920	16,906
Completeness (%)	98.9(97.3)^a^	99.0 (97.3)	99.0 (98.2)	89.7 (53.1)	99.0 (94.0)	89.7 (53)	99.9 (99.9)	99.4 (96.7)	99.4 (97.2)
Mean I/σ(I)	12.6 (1.8)^a^	12.3 (1.7)	12.8 (1.8)	11.2(2.2)	12.5(2.5)	12.6(2.1)	12.0(2.2)	12.1(2.3)	11.5(2.3)
R_merge_ on I (%)	5.2 (87)^a^	5.5 (89)	5.4 (75)	5.9(40.4)	5.3(47)	5.3 (43.8)	6.4(58.2)	6.4(51.5)	6.9 (53)
Highest resolution shell (Å)	1.52–1.44	1.52–1.44	1.60–1.52	1.37–1.30	1.32–1.25	1.37–1.30	2.27–2.15	2.32–2.2	2.37–2.24
**Model and refinement statistics**									
Resolution range (Å)		29.7–1.44			31.2–1.25		45.6–2.15		
No. of reflections (total)		132,168			93,405		19,284		
No. of reflections (test)		6,661			4,682		986		
Cutoff criteria		|F|>0			|F|>0		|F|>0		
R_cryst_ (%)		16.7			13.2		18.2		
R_free_ (%)		19.0			16.3		21.8		
**Stereochemical parameters**									
Restraints (RMS observed)									
Bond lengths (Å)		0.010			0.013		0.009		
Bond angles (°)		0.74			1.46		0.96		
Average isotropic B-value (Å^2^)		31.6			17.1		47.3		
ESU based on R_free_ (Å)		0.063			0.042		0.173		
Protein residues/atoms		706/5,719			355/3,105		318/2,396		

a Highest resolution shells are in parentheses.

ESU = Estimated Standard Uncertainty in atomic coordinates.

R_merge_ = Σ_hkl_Σ_i_|I_i_(hkl)−<I(hkl)>|/Σ_hkl_Σ_i_I_i_(hkl).

R_cryst_ = Σ_hkl_||F_obs_|−|F_calc_||/Σ_hkl_|F_obs_| where F_calc_ and F_obs_ are the calculated and observed structure factor amplitudes, respectively.

R_free_ = as for R_cryst_, but for 5.0% of the total reflections chosen at random and omitted from refinement.

### Structural description

Analysis of crystal packing of each of the three structures showed that the individual molecules interact weakly with each other in the crystal lattices, with a maximum inter-molecular interface of less than 5% of the overall surface area of a monomer. These interfaces are typical of nonspecific crystal contacts, rather than oligomer interfaces [17]. Therefore, both PIBO and IPPA likely exist as a monomer in solution, which is further supported by the results from the size exclusion chromatography coupled with static light scattering (data not shown).

**Figure 1:**
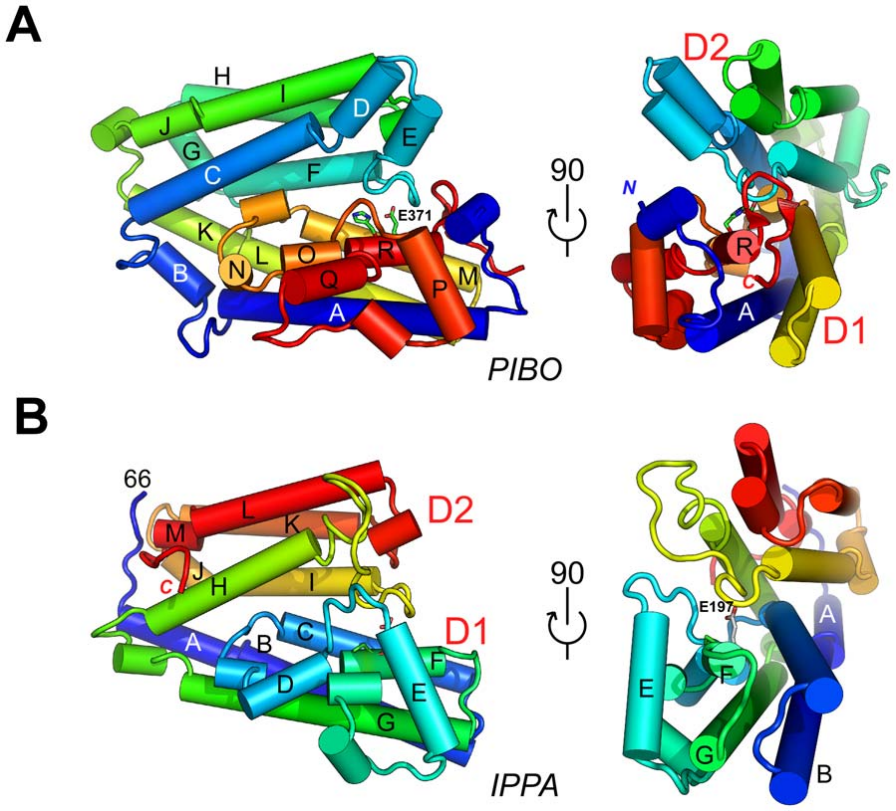
Crystal structures of PIBO and IPPA. (A) Schematic diagram of the structure of PIBO (PDB code 3n8u) color-coded from N-terminus (blue) to C-terminus (red) in two orientations. Helices are represented as tubes and labeled A to R (3_10_ helices are not labeled). His368 and Glu371 of the GxHxxE motif are shown as sticks. (B) Schematic diagram of the structure of IPPA (PDB code 3pf0) color-coded from N-terminus (blue) to C-terminus (red) in the same orientation as PIBO. Helices are represented as tubes and labeled A to M (3_10_ helices are not labeled). Pro194 and Glu197 of the GxxxxE motif are shown as sticks.

The PIBO monomer is a V-shaped, all helical structure consisting of two domains, D1 (residues 33–90 and 244–384) and D2 (residues 91–243), with molecular dimensions of 73 Å×55 Å×42 Å (Fig. 1A, Fig. S2). Both D1 and D2 contain an up-down-up-down, four-helix bundle core with a left-handed twist. In contrast to typical four-helical bundle structures, the helices in the two bundles of PIBO are often disrupted by short insertions as, for example, between helices αA and αB, αI and αJ, and αK and αL. Additionally, a three-helix insertion (two α-helices and one 3_10_ helix) is found between helices αO and αR that interacts with helix αA and the protein N-terminus. The GxHxxE motif is located on the last helix αR, which is followed by two very short β-strands.

The IPPA overall structure is similar to PIBO with two domains, D1 (residues 71–248) and D2 (residues 249–383) (Fig. 1B, Fig. S3). The GxHxxE motif is located on helix αF, but the histidine is replaced by a proline (Pro194). Additionally, IPPA also contains a disulfide bond between Cys110 and Cys211, which is not conserved.

Domains D1 and D2 are similar in structure, with an rmsd of 3.63 Å for 108 superposed Cα atoms between the two domains of IPPA (seq id 5%), and 3.9 Å for 102 Cα atoms between the equivalent two domains of PIBO (seq id 9%). Therefore, imelysin-like proteins may have evolved through gene duplication of an ancestral four-helix bundle.

### PIBO is related to other imelysin-like proteins by circular permutation

**Figure 2:**
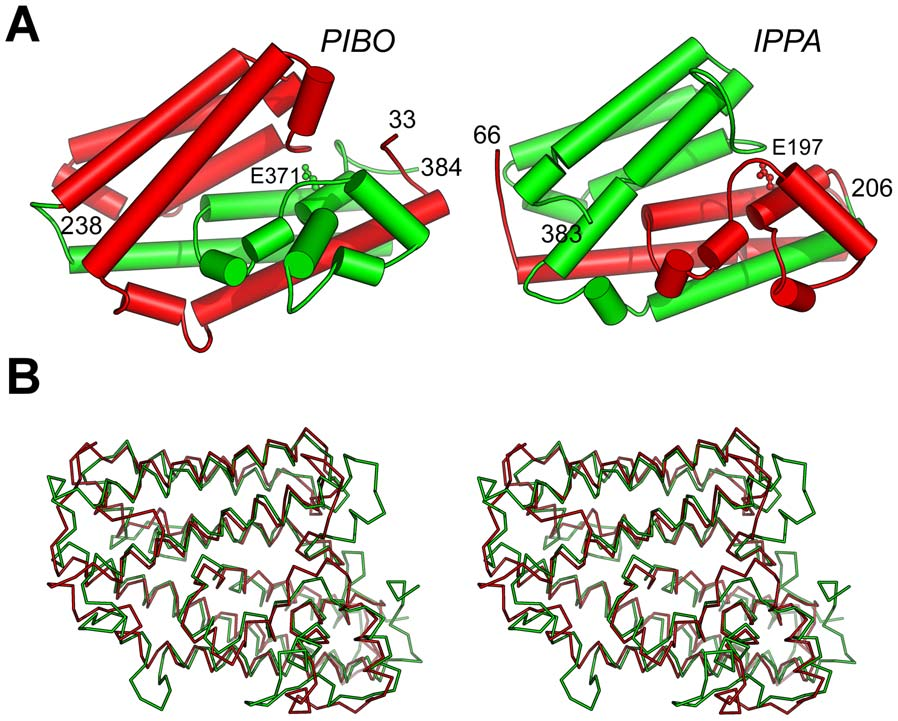
PIBO is related to IPPA by circular permutation. (A) PIBO and IPPA are colored according to circularly permuted fragments. N-terminal and C-terminal fragments are colored red and green respectively. The side chains of the conserved glutamate (Glu 371 and Glu 197) of the GxHxxE motif are shown as ball-and-stick models. (B) Structural comparison between PIBO (green) and IPPA (red), shown in a stereo view.

A BLAST [18] search using PIBO as a probe indicated that PIBO is related to other imelysin-like proteins by a circular permutation, such that the N-terminal (residues 40–237) and C-terminal (238–384) regions are swapped in the primary sequence compared to other imelysin-like proteins, such as IPPA (Fig. 2A). The circular permutation results in the placement of the GxHxxE motif at the C-terminus of PIBO, instead of in the middle of the protein as observed in ICMP and IrpA. Permuted imelysin-like proteins are only found in Bacteriodales (e.g. *B. ovatus* ATCC 8483, *Bacteroides thetaiotaomicron* VPI-5482, *Bacteroides eggerthii* DSM 20697 and *Prevotella bergensis* DSM 17361), and are highly similar to each other, sharing more than 50% sequence identity. A subset of Bacteriodales contains canonical imelysin-like proteins, which are closely related to the permuted proteins. For example, PIBO shares 45% sequence identity with BDI2603 from *Parabacteroides distasonis* ATCC 8503. Thus, these data suggest that circular permutation of imelysins in Bacteriodales is a more recent evolutionary event.

The predicted circularly permuted topology of PIBO is clearly confirmed by comparing its structure to that of IPPA (Fig. 2A), which has a canonical imelysin fold. These structures are highly similar to each other with an rmsd of 2.6 Å for 304 equivalent Cα atoms (seq id 13.2%, Fig. 2B). Moreover, the spatial arrangement of conserved residues (such as the GxHxxE motifs) at the domain interface is also not affected by the circular permutation (see below).

### Structural comparisons

We performed DALI [19] searches using PIBO and IPPA structures as probes to find similar structures. The top hits include other proteins containing four-helix bundles, which are abundant in nature and possess diverse functions. The best hit is a hypothetical protein PF0695 (PDB code 3cax, Z = 8.8), which superposes onto PIBO with an rmsd of 2.7 Å for 150 aligned Cα atoms in the four-helix bundle of D2 (seq id 8%). An intriguing entry in the top hits is cytochrome *b*, which has an overall shape that is similar to that of PIBO and IPPA. PIBO can be superposed on the P chain of the cytochrome *bc*1 (PDB code 3h1j) [20] with an rmsd of 4.4 Å for 204 aligned Cα atoms (Z = 8.1, seq id 5%). Similar regions include six helices within the two four-helix bundles. However, PIBO has a hydrophilic molecular surface and is soluble in water, while cytochrome *b* is an integral membrane protein. As a result, the structural similarity between PIBO (or IPPA) and cytochrome *b* does not appear to implicate an evolutionary or functional connection. We also performed DALI searches using individual domains, but none of these top hits appears to have functional relevance either. Therefore, we conclude that the overall structures of PIBO and IPPA are novel.

The all-helical structures of PIBO and IPPA are distinctive from iron-binding periplasmic binding proteins (PBPs), which consist of two α/β domains [21]. Also, PIBO and IPPA do not share overall structural similarity with any known peptidases. To our knowledge, there are no known peptidases with an active site on the surface of a four-helix bundle.

### Domain interface and putative functional site

**Figure 3:**
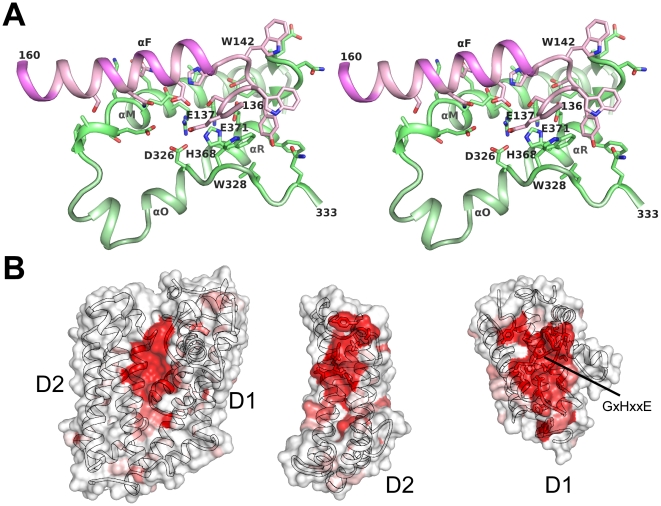
Domain interface of PIBO. (A) Stereo view of the interface between domains D1 (green) and D2 (pink). Residues at the domain interface are shown as sticks. (B) Mapping of the degree of sequence conservation onto the surface of the PIBO structure. The cross-sectional views of each domain interface are shown. The sequence conservation is illustrated by a color gradient from white (not conserved) to red (highly conserved), and was calculated based on 30 non-permuted homologs with best BLAST scores to PIBO.

The interactions between the two domains of PIBO are primarily mediated by helical interactions between helix F of domain D2 and helices αM, αO and αR of domain D1 (Fig. 3A). Additionally, the loop between helices αE and αF and the C-terminal short β-hairpin (after helix R) interact with each other. The interface buries an area of ∼2630 Å^2^ (∼1315 Å^2^ per domain) with six tryptophan residues near the interface (Trp69, Trp139, Trp142, Trp298, Trp305, and Trp328). However, the overall interface is dominated by polar interactions involving multiple hydrogen bonds and salt bridges, such as between Asp147 and Arg302, His143 and Glu306, Lys116 and Glu309, Ser141 and Glu371, Glu137 and His368, and Asn150 and Glu306.

The functional site of PIBO is likely located at the domain interface and supported by the clustering of conserved residues, including the buried GxHxxE motif (Fig. 3B). A small pocket near His368, formed by Glu111, Lys116, Glu137, Asp147, Asn151, Asp326, and Asp309, could serve as a potential active site or substrate-binding site for a small molecule. Water and solvent molecules from the crystallization reagents in both crystal forms occupy this site. In crystal form 1, a magnesium ion, which is present in the crystallization solution, is found at the mouth of the pocket. However, it is coordinated by water molecules only, and does not directly interact with any side-chain atoms. In crystal form 2, a glycerol is found in place of the magnesium. Since these interactions are not specific, we do not consider these ligands physiologically relevant.

**Figure 4:**
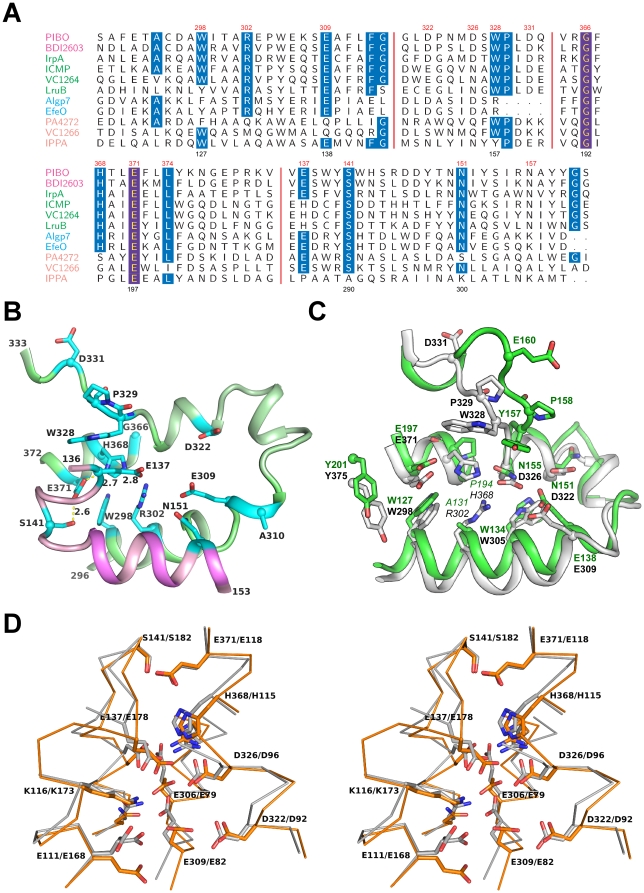
Putative functional site. (A) Most highly conserved regions of a multiple sequence alignment of PIBO, IPPA and other partially characterized homologs. The residue numberings for PIBO and IPPA are shown at the top and bottom rows, respectively. All proteins shown, except PIBO, have a canonical imelysin fold. Protein names are colored by families as shown in Fig. 7. (B) Close-up stereo-view of highly conserved residues in PIBO. (C) Structural comparison of the putative functional sites of PIBO (white) and IPPA (green). (D) Stereo view of the structural comparison of the putative functional sites of PIBO (white) and Algp7 (orange, PDB code: 3at7). Side-chains of strictly conserved residues are shown as sticks: PIBO: Glu111(Algp7:Glu168), Lys116(Lys173), Glu137(Glu178), Ser141(Ser182), Aspp147 (Asp188), Asn151(Asn192), Glu306 (Glu79), Arg302(Arg75), Glu309(Glu82), Asp322(Asp92), Asp326(Asp96), His368(His115), and Glu371 (Glu118).

**Figure 5:**
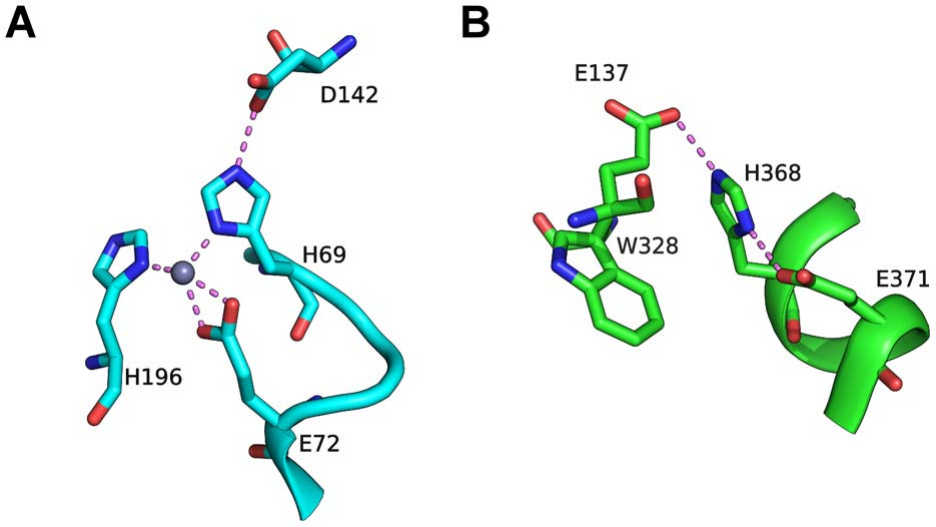
Comparison of the HxxE motifs in carboxypeptidase A [(A), PDB code 3cpa)] and PIBO [(B), PDB code 3n8u)]. The two structures are shown using as similar orientations as possible for the common histidines. Zinc is shown as a gray sphere.

The conserved residues (including the GxHxxE motif) at the domain interface of PIBO are similar to other known imelysin-like proteins that are regulated by iron, such as ICMP, IrpA and EfeO (Fig. 4A–B). These residues are mostly located on a U-shaped, helical hairpin within domain D1 (helical residues 296–333, 366–372). Only a couple of conserved residues are located on domain D2 (Ser141, and Asn151). The side chains of Glu137, His368, Glu371 and Ser141 form a hydrogen bond relay system. His368 and Glu371 of the GxHxxE motif were speculated to be involved in binding divalent metals. Mg^2+^ was present in the crystallization buffer; however, neither residue is involved in the binding of the Mg^2+^ identified in the crystal structure. This lack of observed metal binding in this region, despite its presence in solution, casts doubt on PIBO's ability to bind divalent ions directly. Glu137 seems the only possible candidate to function as a third ligand for metal complexation due to its proximity to the putative functional site (replaced by His in ICMP and LruB). However, these residues are arranged differently from other known HxxE metallopeptidases (Fig. 5). Moreover, the GxHxxE motif of PIBO is located on a short helix, while the metallopeptidase HxxE motif is within a turn in carboxypeptidase A [22]. These results are consistent with thermofluor binding assays, which showed that PIBO did not bind zinc (data not shown).

We searched the structural database for local structural similarity using different combinations of conserved residues near GxHxxE motif (e.g. Glu137, Ser141, His368 and Glu371) [23] and did not identify any other peptidases or other enzymes with a similar arrangement of “active site” residues. As a result, our structural evidence clearly argues against PIBO being a metallopeptidase. Due to the conserved nature of the GxHxxE motif and surrounding regions in imelysin-like proteins (Fig. 4), it is unlikely that any imelysin-like protein functions as a metallopeptidase, unless the peptidase active site is located elsewhere. ICMP contains a lipoprotein signal peptide, and is expected to be an extracellular, soluble protein. The previous characterization of ICMP as a membrane metallopeptidase could be due to contamination, since ICMP was likely denatured by the detergent used during its purification [5].

The putative substrate-binding site of IPPA is very similar to PIBO with two important differences (Fig. 4C). The conserved residues between IPPA and PIBO are all located on D1. The highly conserved serine (Ser141) in D2 is replaced by an alanine in IPPA. Furthermore, among the conserved residues in D1, the residues located in the center are not conserved with substitution of His368 by Pro194, and Arg302 by Ala131. IPPA is also not stabilized by zinc in thermofluor assays; indeed, addition of zinc has a significant destabilizing effect.

During preparation of this manuscript, the crystal structure of the Algp7 protein from *Sphingomonas sp. A1* was published [24]. The Algp7 structure is similar to PIBO (rmsd of 2.3 Å for 251 aligned Cα atoms, seq id 18.6%) and IPPA (rmsd of 2.4 Å for 242 aligned Cα atoms, seq id 10.3%) presented here, despite low sequence identity. The functional sites of Algp7 and PIBO are highly conserved (Fig. 4D).

### Potential functional role of a mobile region

**Figure 6:**
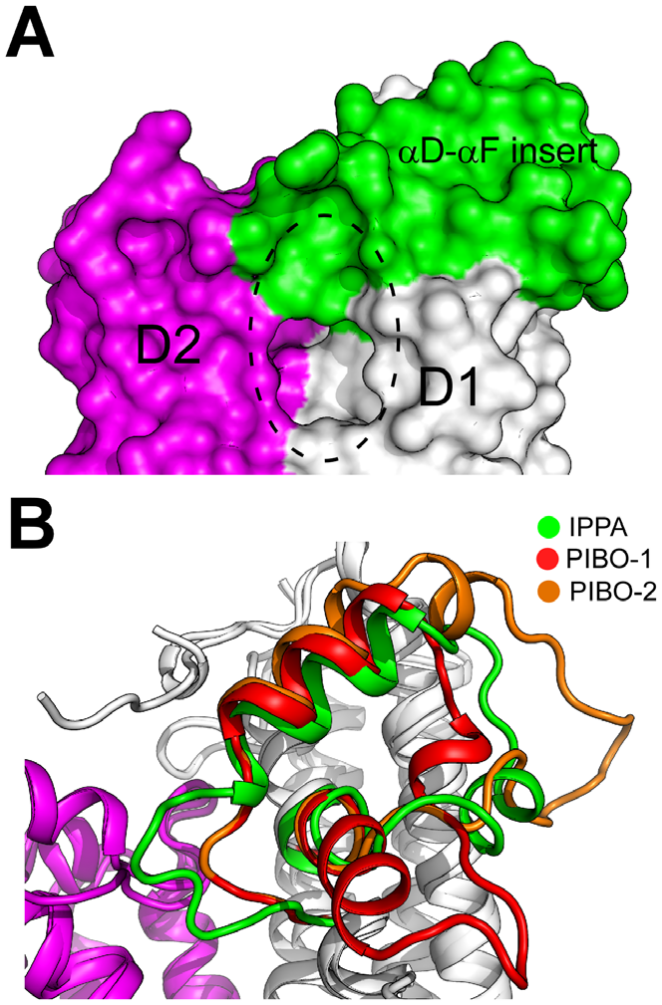
Structural flexibility of PIBO. (A) Surface representation of the αD-αF insert of IPPA (green) with respect to domains D1 (white) and D2 (magenta). (B) Structural comparisons of the αD-αF (IPPA) or αO-αR (PIBO) inserts between IPPA (green) and two crystal forms of PIBO (red, crystal form 1, PDB code 3n8u; orange, crystal form 2, PDB code 3oyv).

A unique structural feature near the putative functional site is the region between two coaxially aligned helices, αD and αF in IPPA (αO and αR in PIBO). The second helix starts with the invariant glycine of the GxHxxE motif, enabling this helix to align and pack tightly with the first helix. An insert between the two helices is located at the entrance the putative functional site and helps shield the domain interface (Fig. 6A). Interestingly, significant structural flexibility is observed in this region. The αO-αR insert (defined as the region between αO and αR) of PIBO adopts different conformations in the two crystal forms. The overall conformation of the αD-αF insert in IPPA is structurally more similar to that of crystal form 2 of PIBO (Fig. 6B). The αO-αR insert in crystal form 1 contains two helices (αP and αQ), as well as a short 3_10_ helix. Helix αQ (residues 355–362) interacts with helix αO, while the 3_10_ helix and the loops connecting to it lie in the interface between helix A and helices αO and αR. However, helix αQ is unwound before residue 361 in crystal form 2, where the peptide changes its direction towards helix αA (Fig. 6B). These observed conformational changes are likely influenced by differences in crystal packing and environment. This insert is likely to be flexible in solution, which could be an important factor for regulating the access to the functional site.

The middle portion of helix αI (residues 215–231) in the second crystal of PIBO displays discrete structural heterogeneity and a helical shift (Fig. S4). The two conformers differ by a main-chain shift, resulting in Cα displacements that vary between 0.6 Å and 1.5 Å. The conformation of equivalent regions in crystal form 1 is intermediate between the two states described above (Fig. S4). Thus, it appears that this region fluctuates between different sub-states, with its conformation affected by crystal packing or other external factors. Such a helical shift within a stable helical bundle was observed previously in the high-resolution structure of the phosphotransfer domain of CheA [25].

### Structural and functional relationship of the imelysin-like proteins

**Figure 7:**
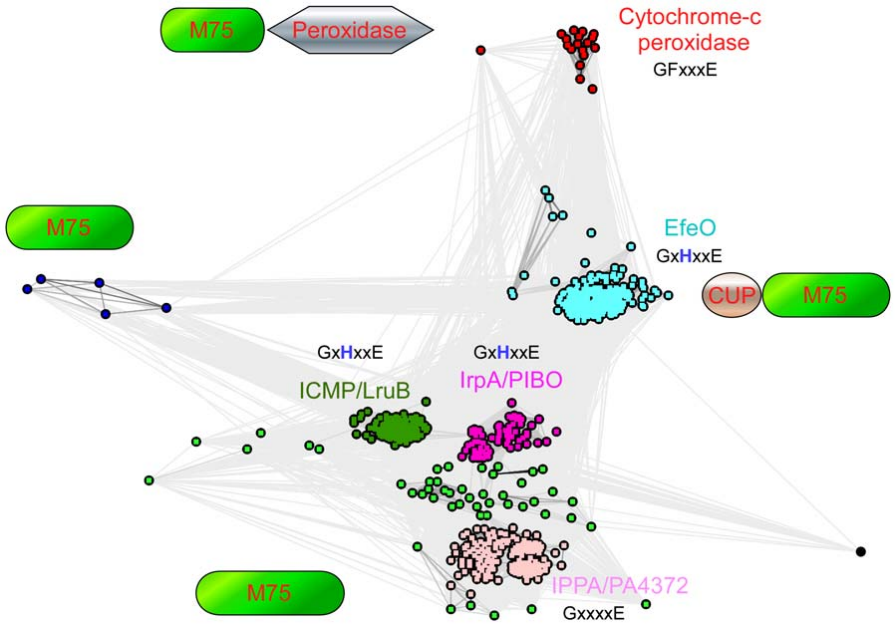
Two-dimensional projection of the CLANS clustering results obtained for the full-length sequences of the imelysin-like proteins. Individual proteins are represented by dots, colored according to the membership in different families or subfamilies. Lines indicate sequence similarity detectable with BLAST and are colored by a spectrum of shades of grey according to the BLAST P-value (black, P-value<10^−200^; light grey, P-value<10^−5^). Imelysin-like proteins cluster into 4 main families: EfeO family (cyan), imelysin family (ICMP/LruB; green), IrpA/PIBO family (magenta), and IPPA/PA4372 family (pink). Schematic diagrams of the domain organization and the sequence motif for the GxHxxE region of each family or subfamily are also shown.

To explore the structural and functional relationship of the imelysin-like proteins, ∼800 unique sequences were gathered from NCBI non-redundant (nr) database, by combining multiple PSI-BLAST [18] runs using ICMP, the imelysin-like domain of EfeO, and BDI2603 as search probes (E<0.001). We analyzed these sequences using the CLANS program, which clusters a set of protein sequences using the P-values of high-scoring segment pairs (HSPs) obtained from an all-to-all BLAST search using a version of the Fruchterman-Reingold graph layout algorithm [26]. The clustering result is shown in Fig. 7. These proteins define four main families: imelysin family, IrpA family, IPPA family, and EfeO family.

The imelysin family, represented by ICMP and LruB, consists currently of 186 members. The closely related IrpA family (64 members) consists of proteins mainly from Bacteriodes, γ-proteobacteria and cyanobacteria. This family also includes the Bacteriodes proteins with a permuted topology. These two families, which largely overlap with the M75 family defined in MEROPS, have a highly conserved functional site. Another family (152 members) is represented by IPPA, and includes *P. aeruginosa* PA4372 and *V. cholera* VC1266. PA4372 or VC1266 is located in the same operon with upstream imelysins (PA4370 or VC1264) and, thus, is also regulated by Fur. They share both the overall fold, as well as a similar functional site, as ICMP. However, the histidine in the GxHxxE motif region is no longer conserved (GxxxxE, Fig. 4A), which may indicate a loss of enzymatic function or a change in substrate preference (compared to imelysin and IrpA families).

Members of the EfeO family (∼350 members) are more closely related to each other, compared to the imelysin family members. They typically contain a CUP domain, in addition to an imelysin-like domain. A few members of this family are fused to an N-terminal putative EfeU ion permease domain. The imelysin-like domain of this family also contains the GxHxxE sequence motif and a highly conserved functional site (Fig. 4A and D), suggesting a similar role as in other imelysin family proteins that contain the same motif.

Most proteins in the superfamily have detectable signal peptides or lipoprotein signal peptides, indicative of non-cytoplasmic localizations. Imelysin-like proteins that are associated with the inner membrane permeases (EfeO) are expected to localize to the periplasm [9]. Most imelysin-like proteins with the GxxxxE motif are predicted to be in the periplasm. A significant percentage of imelysin-like proteins with lipoprotein signal peptides are predicted to be on the outer membrane as, for example, PIBO, ICMP, and LruB.

**Figure 8:**
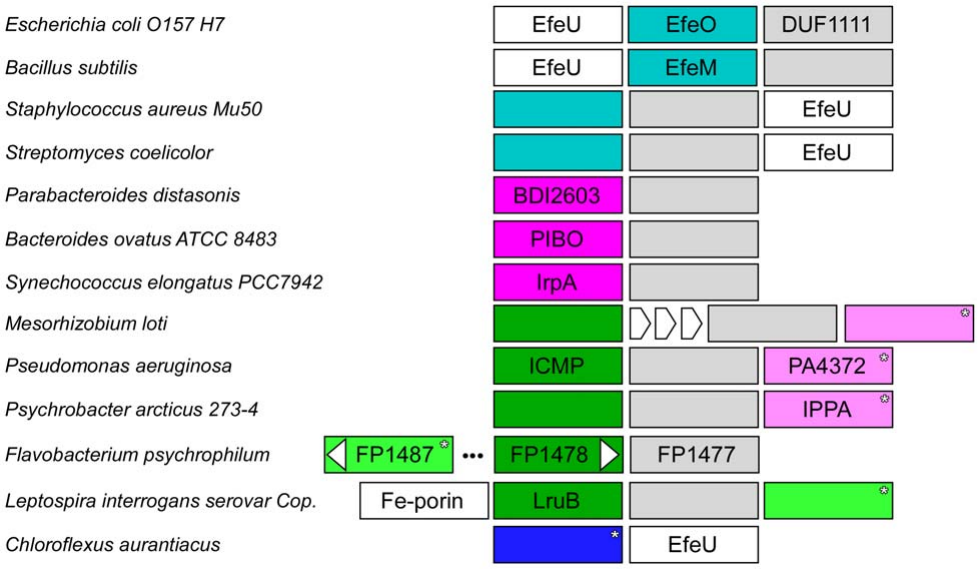
Representative genomic contexts for imelysin-like proteins. Imelysin-like proteins are colored by families as in Fig. 7 (IPPA family: pink, IrpA family: magenta, EfeO family: cyan, ICMP family: dark green, unclassified: blue and light green). Imelysin-like proteins that have a GxxxxE motif are marked by an asterisk. DUF1111 is a family of di-heme peroxidases (colored gray). EfeU belongs to the Ftr1 iron transport protein family. The direction of transcription is from left to right (except for FP1487).

As demonstrated above, structural representatives from three families, PIBO, IPPA and Algp7 (a member of EfeO family), reveal a conserved overall structure and functional site, suggesting that imelysin-like proteins have evolved from a common ancestor. The Efe-like system is widely distributed in other bacteria with highly conserved operons (Fig. 8). Imelysin-like proteins are almost always associated with an EfeB peroxidase in the genome. Thus, the functions of imelysin-like proteins are very likely linked to EfeB-like peroxidases. This functional link is further supported by the existence of a small group of proteins containing an imelysin-like domain fused to an EfeB-like domain (Fig. 7).

### Functional implications

The biochemical functions of imelysin-like proteins are poorly understood. In the EfeUOB system that is involved in Fe^2+^ uptake, it is assumed that the iron-dependent peroxidase EfeB converts ferric iron into ferrous iron, which is then transferred to the EfeU permease by EfeO [9]. However, the detailed biochemical functions and substrate specificity of EfeB and EfeO are currently unknown. The functional sites in PIBO and IPPA do not resemble known enzymes, which appear to be consistent with their putative function as binding proteins. However, the nature of the substrate remains unclear.

Proteins binding iron or iron-containing compounds commonly adopt helical folds, such as helical bundles. However, we were unable to identify a GxHxxE motif in other known iron-binding proteins, which often contain an ExxH motif (e.g. in transferrin). A histidine stabilized by a hydrogen bond from a carboxylate group is often found in heme-binding proteins where the histidine serves as an axial ligand to the iron. The conserved histidine in the GxHxxE motif of PIBO could serve a similar role. As a result, we tested the binding of heme or ferric ion by PIBO and IPPA using the thermofluor method. Addition of heme ligands (hemin or hematin) resulted in decreased melting temperatures, while ferric ion had no significant effect, indicating that neither protein specifically binds these ligands (data not shown). These results are consistent with the crystal structures, which indicate that the binding site is too small to accommodate heme without conformational changes.

Imelysin-like proteins may have functional roles other than binding iron-containing compound(s) directly. For example, they might help to mediate protein complexes between the EfeB-like peroxidases and transmembrane transporters. Those proteins with GxHxxE motif may represent novel enzymes. Interestingly, Algp7 was found to bind alginate at neutral pH [27]. Algp7 shares ∼60% sequence identity to the imelysin-like region of EfeO, but does not contain the CUP domain. An alginate-binding role for the imelysin-like domain of EfeO does not seem to be related to iron uptake. Furthermore, the structures suggest that the binding site for alginate is more likely to be on the protein surface, which is not conserved. Thus, the generality of alginate as a substrate for other imelysin-like proteins needs further investigation.

In summary, the structures presented here offer valuable insights into potential functions of these novel proteins and lay the foundation for further biochemical experiments, which are clearly needed in order to elucidate the detailed roles of PIBO, IPPA and other imelysin-like proteins.

## Materials and Methods

### Protein expression and purification

Clones were generated using the Polymerase Incomplete Primer Extension (PIPE) cloning method [28]. The gene encoding PIBO (Locus name: BACOVA_03801, GI: ZP_02066800.1, UNIPROT: A7M120) was amplified by polymerase chain reaction (PCR) from *B. ovatus* ATCC 8483 genomic DNA using *PfuTurbo* DNA polymerase (Stratagene) and I-PIPE (Insert) primers (forward primer, 5′-ctgtacttccagggcAGTGATGATGACAACCCAACAGTAGATC-3′; reverse primer, 5′-aattaagtcgcgttaTTGTACTTTACGTGGTTCACCGTTTTTG-3′, target sequence in upper case) that included sequences for the predicted 5′ and 3′ ends. The expression vector, pSpeedET, which encodes an amino-terminal TEV protease-cleavable expression and purification tag (MGSDKIHHHHHHENLYFQ/G), was PCR amplified with V-PIPE (Vector) primers (forward primer: 5′-taacgcgacttaattaactcgtttaaacggtctccagc-3′, reverse primer: 5′-gccctggaagtacaggttttcgtgatgatgatgatgatg-3′). V-PIPE and I-PIPE PCR products were mixed to anneal the amplified DNA fragments together. *E. coli* GeneHogs (Invitrogen) competent cells were transformed with the I-PIPE/V-PIPE mixture and dispensed on selective LB-agar plates. The cloning junctions were confirmed by DNA sequencing. Using the PIPE method, the gene segment encoding residues M1-S24 was deleted prior to PCRs. Expression was performed in a selenomethionine-containing medium at 37°C. Selenomethionine was incorporated via inhibition of methionine biosynthesis [29], which does not require a methionine auxotrophic strain. At the end of fermentation, lysozyme was added to the culture to a final concentration of 250 µg/ml, and the cells were harvested and frozen. After one freeze/thaw cycle, the cells were homogenized and sonicated in lysis buffer [50 mM HEPES pH 8.0, 50 mM NaCl, 10 mM imidazole, 1 mM Tris (2-carboxyethyl) phosphine-HCl (TCEP)] and passed through a Microfluidizer (Microfluidics). The lysate was clarified by centrifugation at 32,500×g for 30 minutes and loaded onto a nickel-chelating resin (GE Healthcare) pre-equilibrated with lysis buffer, the resin washed with wash buffer [50 mM HEPES pH 8.0, 300 mM NaCl, 40 mM imidazole, 10% (v/v) glycerol, 1 mM TCEP], and the protein eluted with elution buffer [20 mM HEPES pH 8.0, 300 mM imidazole, 10% (v/v) glycerol, 1 mM TCEP]. The eluate was buffer exchanged with TEV buffer [20 mM HEPES pH 8.0, 200 mM NaCl, 40 mM imidazole, 1 mM TCEP] using a PD-10 column (GE Healthcare), and incubated with 1 mg of TEV protease per 15 mg of eluted protein for 2 hr at ambient temperature followed by overnight at 4°C.The protease-treated eluate passed over nickel-chelating resin (GE Healthcare) pre-equilibrated with HEPES crystallization buffer [20 mM HEPES pH 8.0, 200 mM NaCl, 40 mM imidazole, 1 mM TCEP] and the resin was washed with the same buffer. The flow-through and wash fractions were combined and concentrated to 16.1 mg/ml by centrifugal ultrafiltration (Millipore) for crystallization trials.

IPPA (Locus name: PSYC_1802, GI: YP_265084.1, UNIPROT: Q4FQQ8_PSYA2) was cloned and purified using a similar protocol above. The clone construct contains residues 27–389 (forward primer, 5′-ctgtacttccagggcGATGACAATAACGCAGCAGAGGTAGAC-3′; reverse primer, 5′-aattaagtcgcgttaATCGCCATCGGTACTATTAAAACCCAC-3′). The purified protein was concentrated to 14.6 mg/ml for crystallization.

### Crystallization

PIBO was crystallized using the nanodroplet vapor diffusion method [30] with standard JCSG crystallization protocols [12]. Sitting drops composed of 200 nl protein solution mixed with 200 nl crystallization solution were equilibrated against a 50 µl reservoir at 277 K for 27 days prior to harvest. The PIBO crystal form 1 was obtained with a precipitating solution composed of 0.2 M Magnesium acetate and 20% PEG 3350. Ethylene glycol was added to a final concentration of 10% (v/v) as a cryoprotectant. The crystallization solution yielding the PIBO crystal form 2 consisted of 0.05 M KH_2_PO_4_ and 20% PEG 8000. Glycerol was added to a final concentration of 20% (v/v) as a cryoprotectant.

The crystallization solution yielding the IPPA crystals was composed of 0.2 M MgCl_2_, 30% PEG 4000 and 0.1 M Tris pH 8.5. The plates were incubated at 277 K for 40 days prior to crystal harvest. Initial screening for diffraction was carried out using the Stanford Automated Mounting system (SAM) [31] at the Stanford Synchrotron Radiation Lightsource (SSRL, Menlo Park, CA).

### Data collection, structure solution, and refinement

Two MAD data for PIBO were collected at wavelength corresponding to the inflection, high energy remote, and peak of a selenium MAD experiment at 100 K using Mar CCD 325 detector (Rayonix) at SSRL beamlines 11-1 (crystal form 1, PDB code 3n8u, two molecules per asu) and 9-2 (crystal form 2, PDB code 3oyv, one molecule per asu). The three-wavelength MAD data for IPPA were collected at 100 K using ADSC Quantum Q315 CCD detector at ALS beamline 8.2.2. All data were integrated and reduced using XDS and then scaled with the program XSCALE [32]. The three structures were determined and refined independently. Selenium sites were located with SHELXD [33]. Phase refinement, density modification and automatic model building were performed using autoSHARP [34] and wARP [35]. Further model completion and refinement were performed with COOT [36] and REFMAC [37] of the CCP4 suite [38] or BUSTER-TNT [39]. TLS parameters were refined with each monomer as a rigid body group for crystal form 1 of PIBO and IPPA using BUSTER-TNT, while full anisotropic B-factors were refined for crystal form 2 of PIBO with REFMAC. Data and refinement statistics are summarized in Table 1. Analysis of the stereochemical quality of the model was accomplished using MolProbity [16]. All molecular graphics were prepared with PyMOL (http://www.pymol.org). Multiple sequence alignment was calculated using T-COFFEE (accurate mode) [40]. Atomic coordinates and experimental structure factors have been deposited in the PDB (http://www.rcsb.org) under accession codes 3n8u and 3oyv for PIBO and 3pf0 for IPPA.

## Supporting Information

Figure S1 **Electron density maps for PIBO in stereo view.** Representative section of the experimental density obtained after density modification using the initial MAD phases, contoured at 1.5 σ. The final refined model (PDB code 3n8u) is shown as sticks.
(PDF)

Figure S2 **Secondary structures of PIBO mapped onto its sequence.** The GxHxxE motif and the conserved cysteine of the lipobox motif are denoted as red dots below the sequence.
(PDF)

Figure S3 **Secondary structures of IPPA mapped onto its sequence.** The GxxxxE motif and the conserved cysteine of the lipobox motif are denoted as red dots below the sequence.
(PDF)

Figure S4 **Stereo view of the αI region (residues 215–231) that displays heterogeneity with a slight variation in shift along the helical axis in crystal form 2.** The carbon atoms of the two observed conformations (PDB code 3oyv) are colored as green and cyan respectively. The corresponding section from crystal form 1 (PDB code 3n8u chain A, carbon atoms colored magenta) is also shown.
(PDF)
