# Supplementary figures and images for: Structural and Sequence Analysis of Imelysin-Like Proteins Implicated in Bacterial Iron Uptake
Source: PLoS One. 2011 Jul 25;6(7):e21875. doi: 10.1371/journal.pone.0021875 (PMC3143127; doi:10.1371/journal.pone.0021875)

## Supporting Information

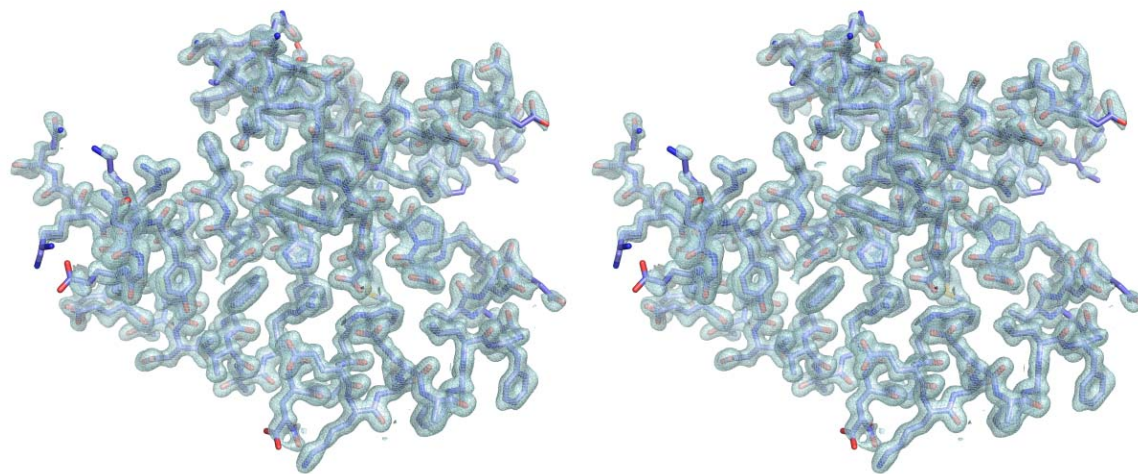

**Fig. S1**

Supplement: Figure S1 — Electron density maps for PIBO in stereo view. Representative section of the experimental density obtained after density modification using the initial MAD phases, contoured at 1.5 σ. The final refined model (PDB code 3n8u) is shown as sticks. (PDF) [file pone.0021875.s001.pdf]

## Supporting Information

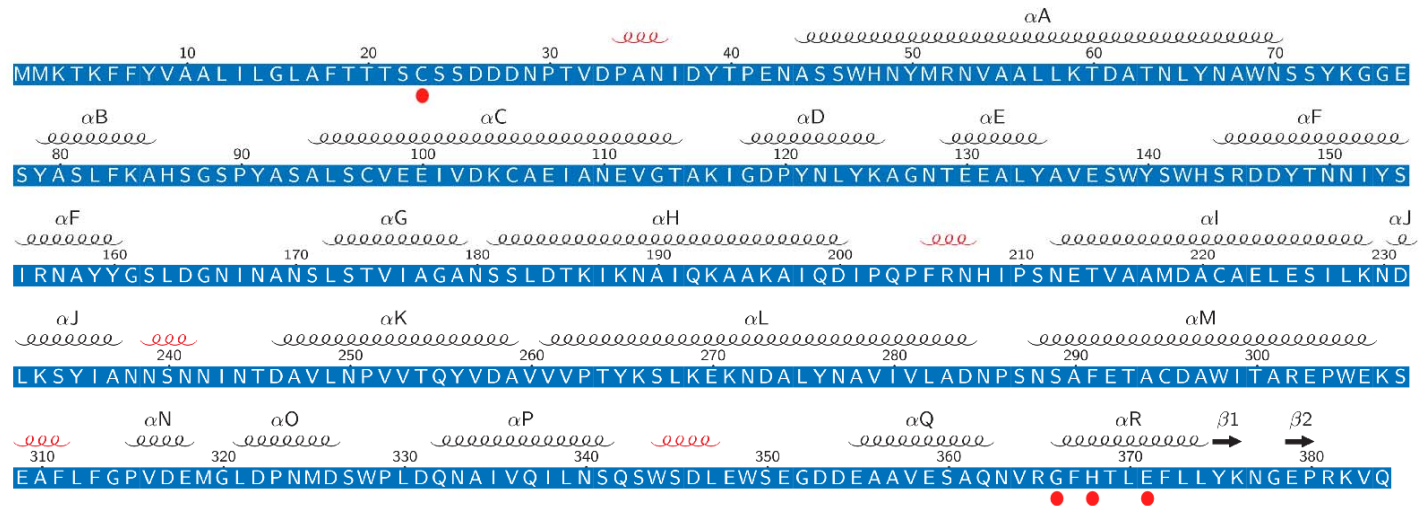

Fig. S2

Supplement: Figure S2 — Secondary structures of PIBO mapped onto its sequence. The GxHxxE motif and the conserved cysteine of the lipobox motif are denoted as red dots below the sequence. (PDF) [file pone.0021875.s002.pdf]

## Supporting Information

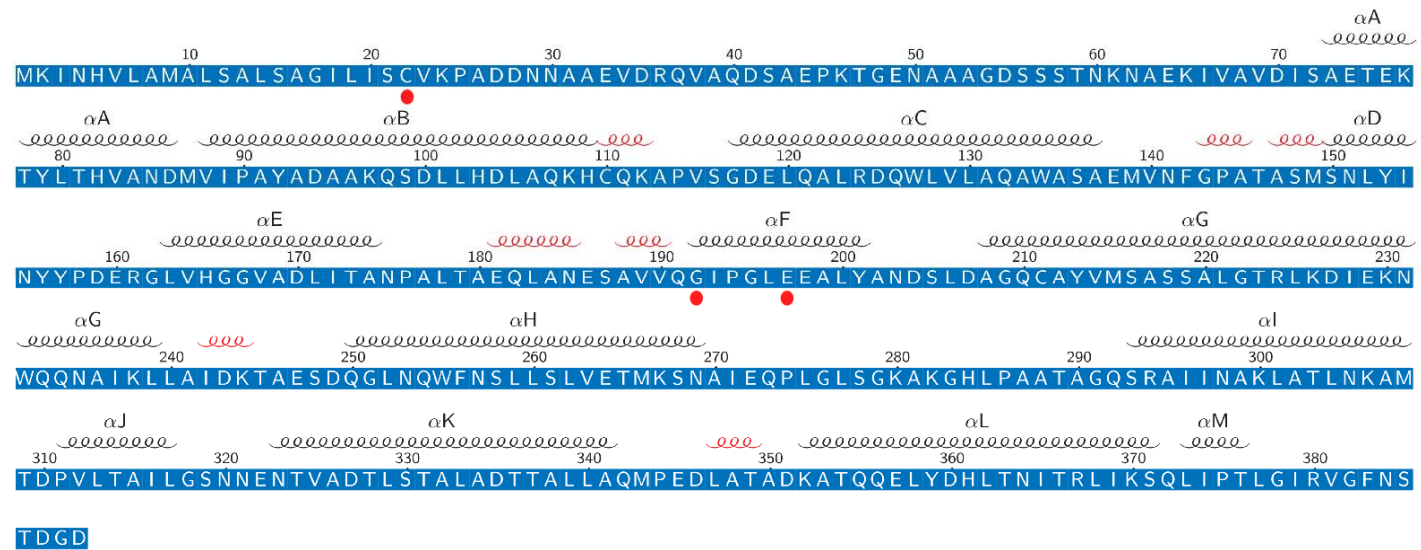

Fig. S3

Supplement: Figure S3 — Secondary structures of IPPA mapped onto its sequence. The GxxxxE motif and the conserved cysteine of the lipobox motif are denoted as red dots below the sequence. (PDF) [file pone.0021875.s003.pdf]

## Supporting Information

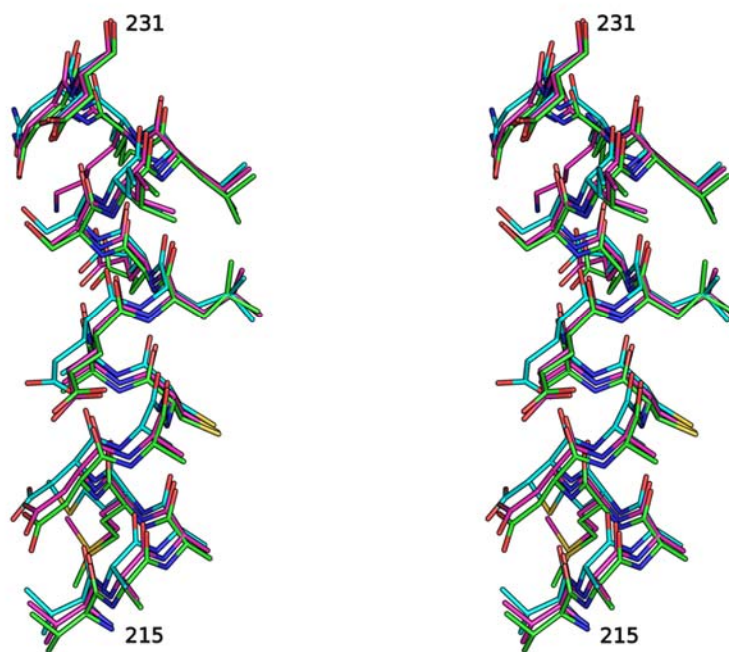

Fig. S4

Supplement: Figure S4 — Stereo view of the αI region (residues 215–231) that displays heterogeneity with a slight variation in shift along the helical axis in crystal form 2. The carbon atoms of the two observed conformations (PDB code 3oyv) are colored as green and cyan respectively. The corresponding section from crystal form 1 (PDB code 3n8u chain A, carbon atoms colored magenta) is also shown. (PDF) [file pone.0021875.s004.pdf]
